# Brachial Artery Injury Resulting From a Dog Bite in a Pediatric Patient: A Case Report

**DOI:** 10.7759/cureus.45889

**Published:** 2023-09-25

**Authors:** Sarinya Meknarit, Adam J Mann, Faris Azar, Robert Borrego

**Affiliations:** 1 Surgery, Florida Atlantic University Charles E. Schmidt College of Medicine, Boca Raton, USA; 2 General Surgery, Florida Atlantic University, Boca Raton, USA; 3 Surgery, St. Mary's Medical Center, West Palm Beach, USA

**Keywords:** saphenous vein graft (svg), trauma, pediatric, upper extremity ischemia, wound exploration, dog bite wounds, animal bite, traumatic vascular injury

## Abstract

Pediatric trauma surgeons frequently encounter severe injuries from animal bites, with dog bites being especially prevalent in children, often leading to facial injuries. This paper details the case of a six-year-old male who suffered a dog bite resulting in a rare proximal right brachial artery injury. The bite caused deep lacerations and avulsion injuries, prompting admission to the trauma center, where nonpalpable right radial and ulnar pulses and arm weakness were observed. Surgical intervention, including wound exploration and brachial artery repair using a saphenous vein graft, successfully restored vascular perfusion. This case underscores the urgency of addressing pediatric dog bite injuries through timely exploration, thorough irrigation, and antibiotic prophylaxis, while also highlighting the need for further research on preventive education and clinical guidelines for assessing vascular injuries in such cases.

## Introduction

Trauma surgery, among other surgical specialties, often bears the brunt of serious injuries from animal bites. The largest subset of these injuries from 1971 to 2018 has been bites from pet canines [1] and is more prevalent in children than adults [2]. In fact, dog bites are among the leading causes of non-fatal emergency room visits in children. Dog bite injury in pediatric patients often results in facial injuries [3], causing both physical and psychological distress [4].

The concerning feature of dog bite injuries is the deep penetration of the puncture wounds, causing injuries to deeper structures including the vasculature. Though vascular injury from a dog bite is uncommon [5], its consequence can be fatal resulting in tissue ischemia if not treated emergently. We present a rare case of a dog bite injury to the proximal right arm in a six-year-old male pediatric patient, resulting in loss of distal perfusion due to an intimal injury and thrombosis of the proximal right brachial artery.

## Case presentation

**Figure 1:**
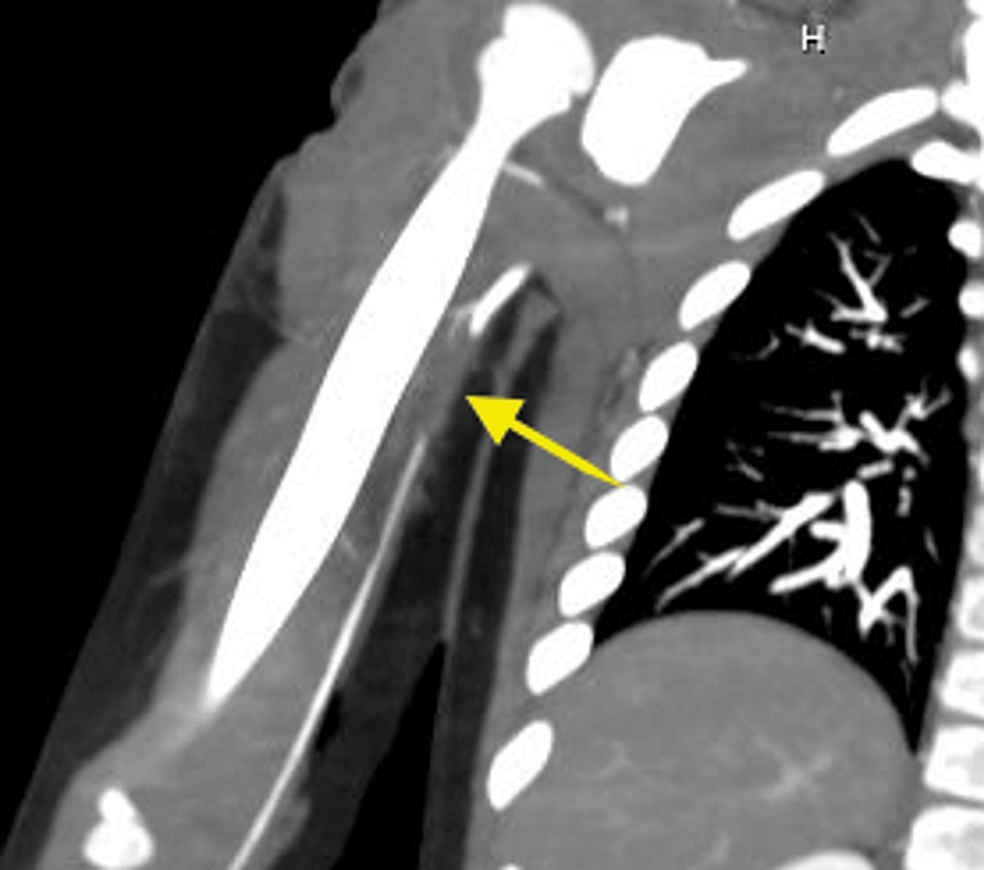
Proximal right brachial artery occlusion without evidence extravasation shown on CTA. The occlusion is 2.5cm in length with reconstitution of the distal brachial artery.

The patient is a 6-year-old male who presented to the trauma center three hours after he was bitten on his proximal right upper extremity by his family dog, a Belgian Malinois, a breed more commonly used in police and military force. Upon examining the wound, there are deep lacerations and avulsion injuries extending through the muscle layer on the medial and lateral upper right arm and within the axilla. Right radial and ulnar pulses were nonpalpable and not detected using Doppler ultrasonography. He had right arm weakness, but motor and sensory function were intact. Computed tomography angiography (CTA) showed evidence of a proximal right brachial artery thrombosis with no extravasation (Figure 1), and multiple soft tissue avulsion injuries of the upper arm and axilla. The patient was given appropriate antibiotic coverage with ceftriaxone and taken directly to the operating room for right upper extremity wound exploration, repair of brachial artery, and wound washout.

**Figure 2:**
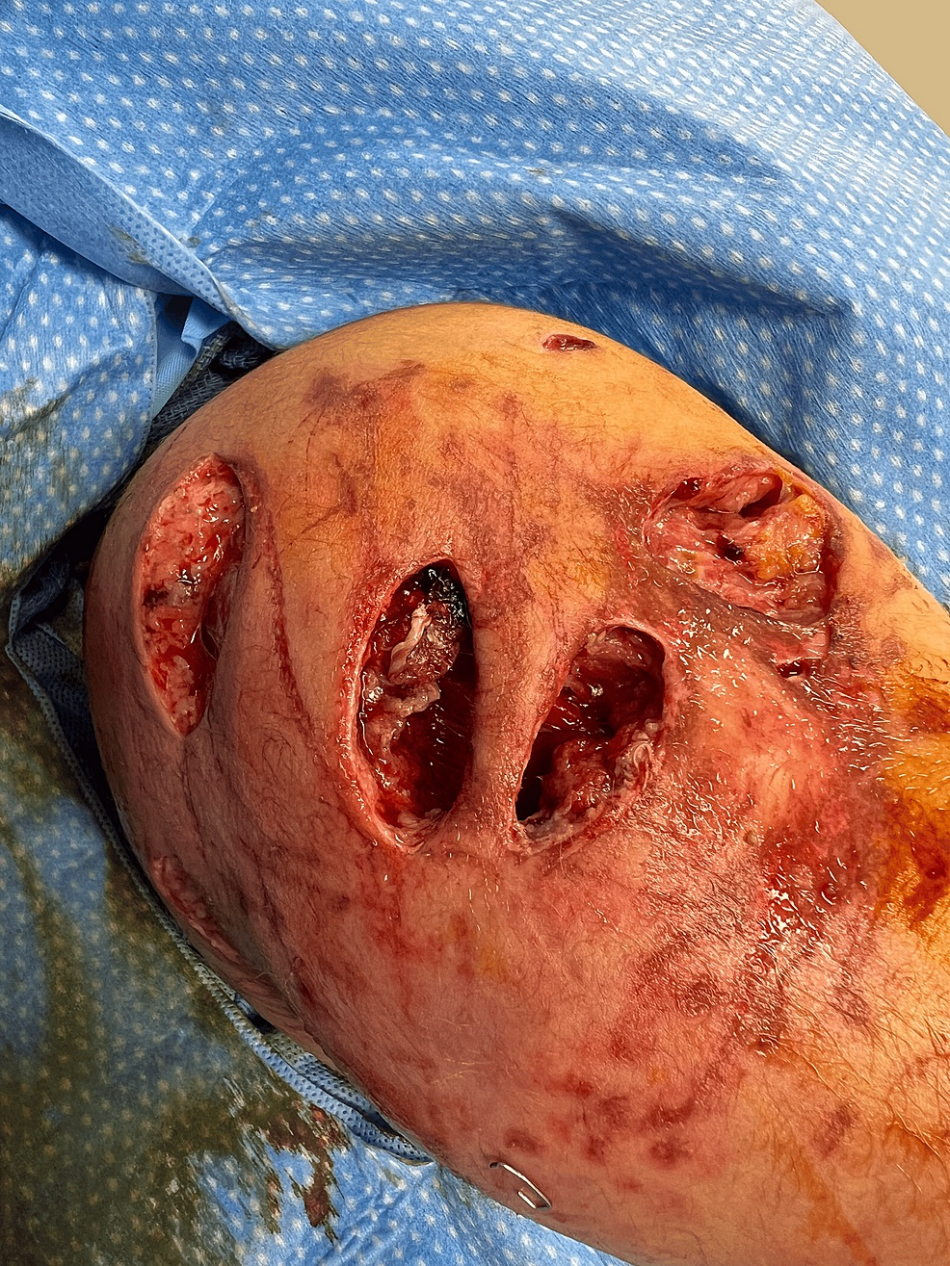
Right arm lateral avulsion injuries measuring 6x4cm, 4x3cm, and 4x2cm down to muscle layer.

**Figure 3:**
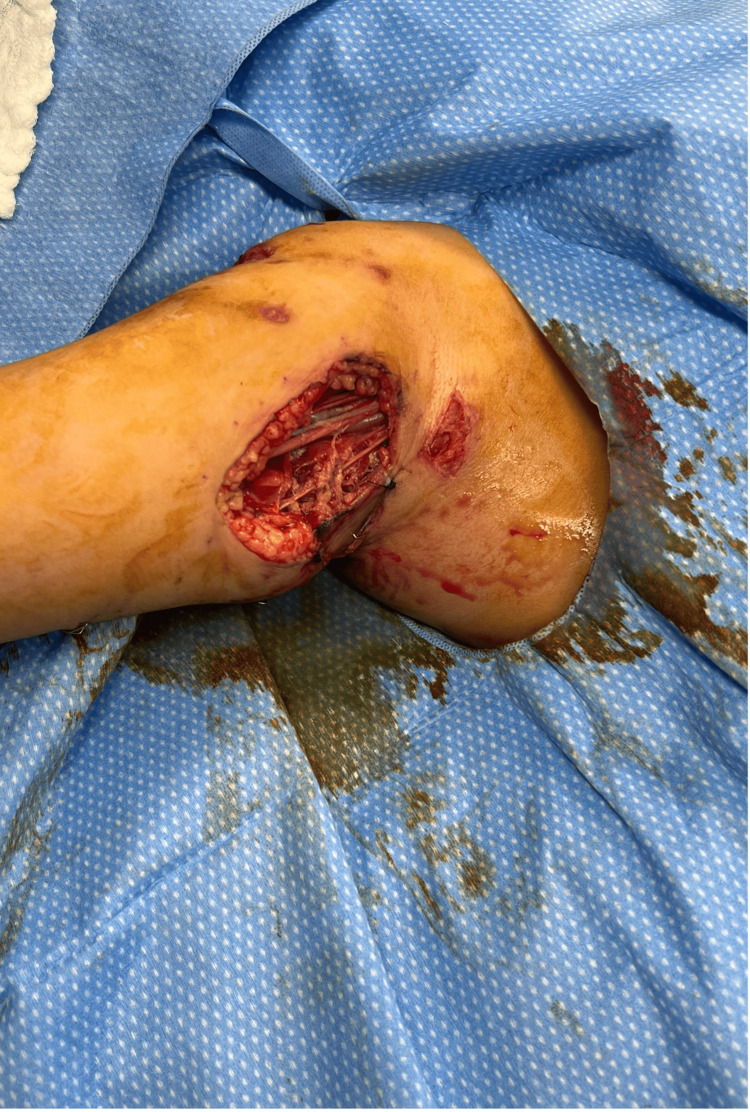
Right arm medial avulsion injuries measuring 7x3cm and 4x2cm down to the muscle layer.

In the operating room under general anesthesia, we identified lateral avulsion injuries measuring 6x4cm, 4x3cm, and 4x2cm down to the muscle (Figure 2), and two medial avulsion injuries measuring 7x3cm and 4x2cm down to the muscle layer (Figure 3). We identified a contused brachial artery with intraluminal thrombosis and no perfusion distally, concerning an intimal injury. 

**Figure 4:**
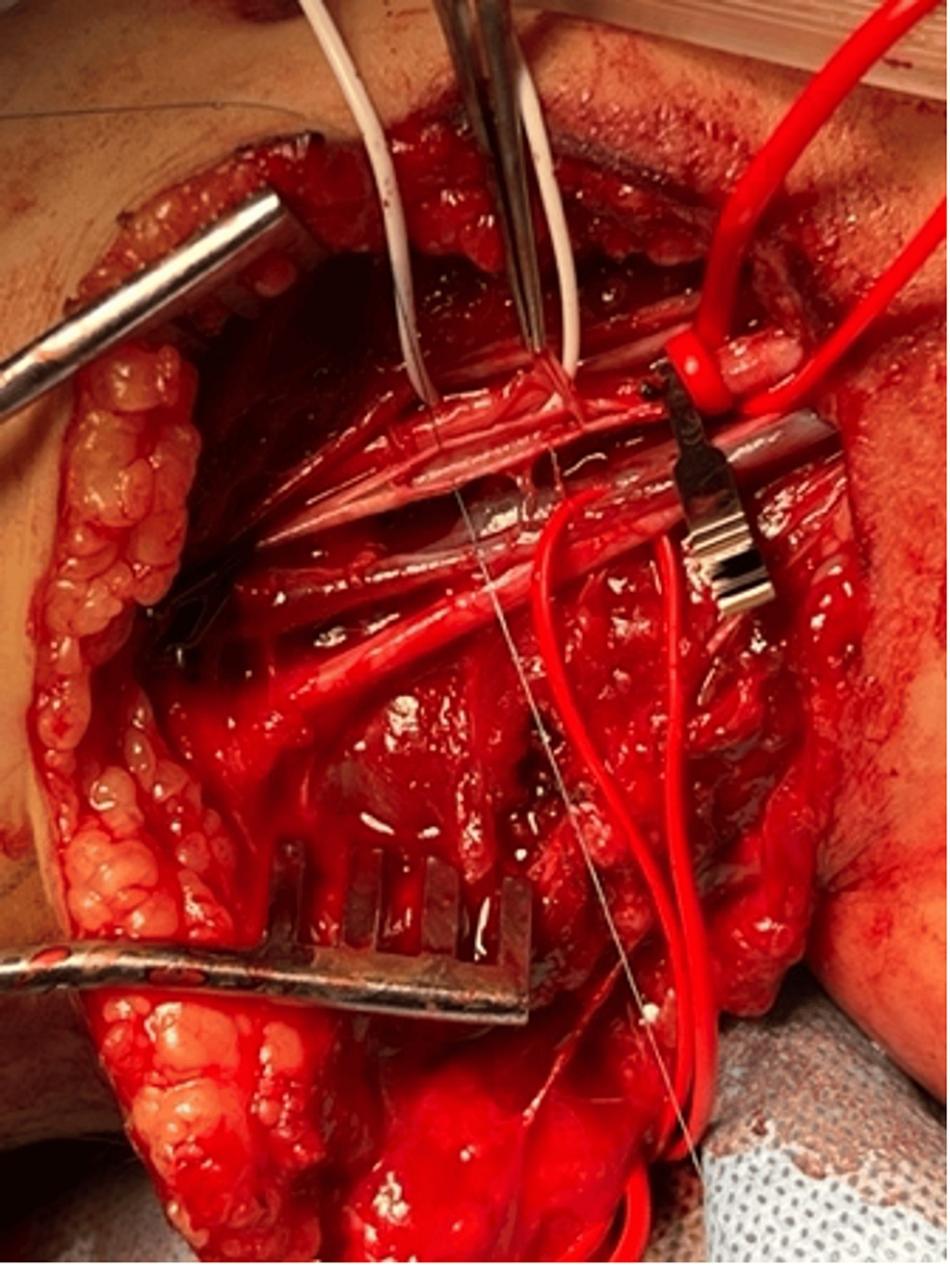
Proximal and distal brachial artery vascular control was obtained in the operating room.

**Figure 5:**
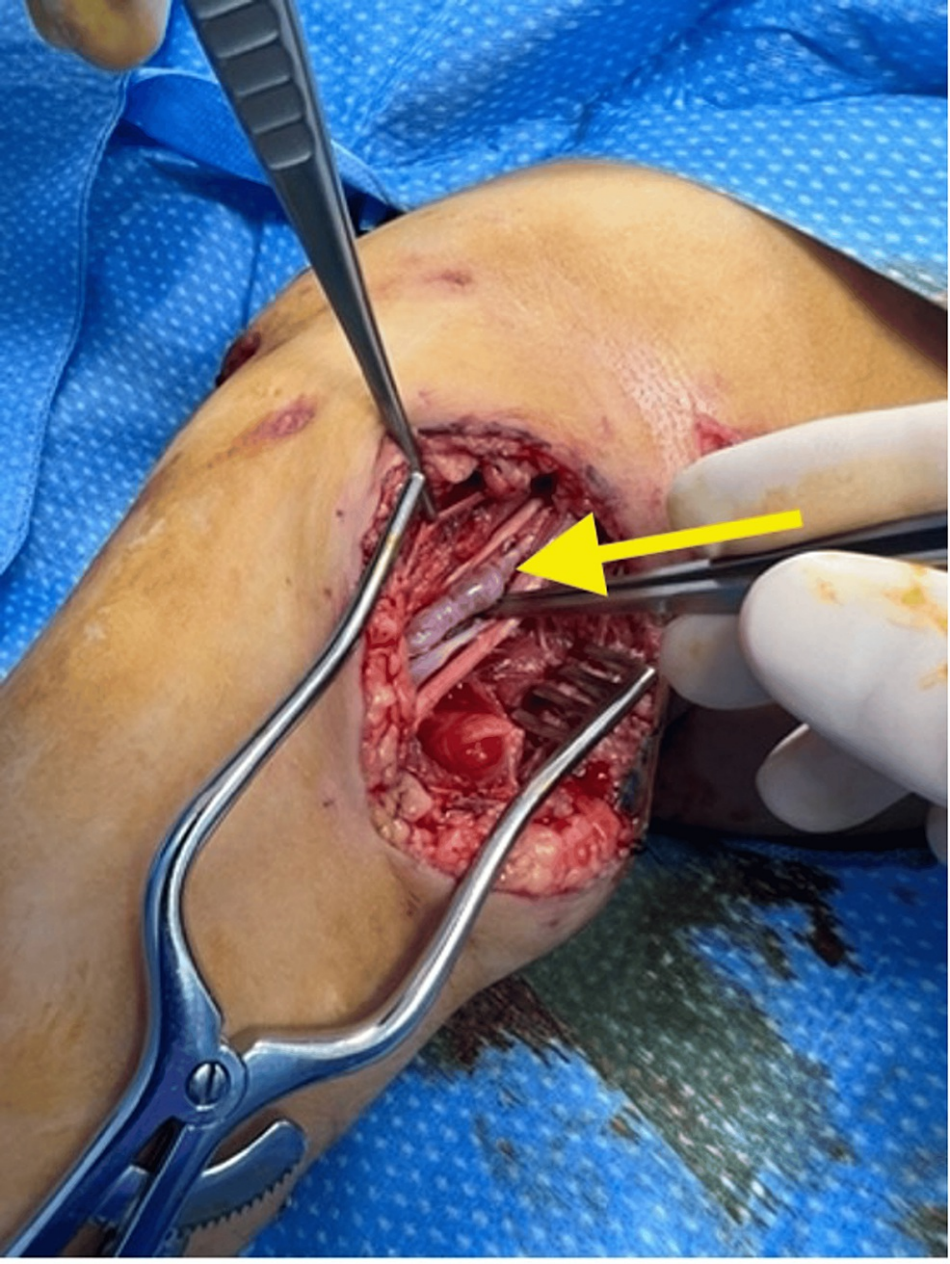
Saphenous vein venous patch anastomosis of the brachial artery.

We simultaneously turned our attention to the right groin for vein harvest. The right saphenous vein was identified using an ultrasound and harvested. The patient was heparinized with 100 units/kg of unfractionated heparin, and proximal and distal vascular control was obtained (Figure 4). A 2-cm anterior arteriotomy was performed followed by endarterectomy, and balloon thrombectomy proximally and distally, which restored both inflow perfusion and back-bleeding. The intimal flap injury was anchored to the vessel wall with 9-0 prolene, and a saphenous vein venous patch anastomosis was performed with 7-0 prolene in a running fashion (Figure 5). Upon completion of the anastomosis, radial and ulnar pulses were palpable, with triphasic doppler signals to both the radial and ulnar arteries. The wounds were heavily irrigated and partially closed to allow drainage. Patient was extubated and monitored for 24 hours in the pediatric intensive care unit for serial vascular exams.

The patient’s hospital course was unremarkable with no fevers, chills, wound infection, leukocytosis, or signs of vascular compromise. The patient was taken subsequently three times (hospital day 2, 4, and 6) to the operating room for washout, debridement, and closure of all wounds by tertiary intention. Patient worked extensively with physical and occupational therapy resulting in dramatic improvement in right upper extremity function. The patient completed a one-week course of antibiotic therapy with Amoxicillin-Clavulanate and was discharged on hospital day 9.

## Discussion

Over 750,000 dog bite injuries result in emergency department visits, with 1%-2.5% of which requiring hospitalization. Children are at higher risk of dog bites than adults, with the highest incidence occurring in children of five to nine years old [6]. They are commonly single-bite wounds, but multiple wounds are also possible as a result of mauling [7]. A concerning feature of dog bites is the depth of injury resulting in injury to the deeper structures such as arteries and muscles. This is especially true with larger dogs and younger children. As shown in our case, this can result in vascular compromise and ischemia if not treated promptly. Significant complications associated with dog bites are rabies, wound infections, post-traumatic stress disorder, and various manifestations of fear and anxiety [7].

The incidence of rabies is minimal in industrialized countries due to the accessibility of post-exposure rabies prophylaxis and canine rabies vaccination. When encountering a patient with a dog bite, physicians should promptly inquire about the patient's rabies vaccination status and the health status of the involved dog, and assess the patient’s need for rabies post-exposure prophylaxis according to the guidelines provided by the CDC [8].

Currently, there is a lack of established clinical guidelines to determine when dog bite wounds should be explored for vascular involvement in patients with normal distal pulses. A retrospective study [9] comparing dog bite injuries with and without vascular injury did not identify any reliable predictive risk factors. This includes factors such as the Glasgow Coma Scale (GCS) upon admission, blood pressure, injury severity score, and the specific type of injury sustained. Based on limited literature, the axillobrachial artery was the most commonly injured followed by the radial artery, with diminished pulses as the most common clinical presentation [4,10]. From our case, we recommend considering surgical wound exploration when there is difficulty visualizing the wound, abnormal Doppler exam, delayed capillary refill, or nonpalpable pulses [11].

Wound infection is the primary complication and sequelae of dog bites. Organisms involved include both aerobic and anaerobic bacteria, and a few fungi [7]. Early and frequent aggressive wound irrigation is recommended to prevent secondary infection, as well as cleaning the area for wound examination [12]. Antibiotics used for prophylaxis should cover a broad spectrum of organisms given the types of microbes found in dog oral flora. Amoxicillin-clavulanate is generally considered a first-line prophylactic treatment for animal bites. We started our patient on a seven-day course of amoxicillin-clavulanate given the severity and depth of the wound. Since hospitalization, the patient had no signs of infection (no fever, chills, purulent drainage from the wound, or leukocytosis).

Wound closure of dog bites is considered contraindicated given the risk of infection. However, there have been studies that showed no significant difference in infection rate between healing by primary and secondary intention [12,13]. However, certain cases may warrant partial closure to promote healing. Thus, we believe that the decision should be based on the severity of the wound. In our case, given the exposure of deeper structures from the bite and the degree of avulsion injury, we partially closed the wound to approximate the skin while also allowing for drainage. The wound was partially closed during each subsequent washout, with all wounds closed by the last washout on hospital day eight. This allowed for the prevention of infection as well as a satisfactory cosmetic outcome.

Most literature studying dog bite injuries is focused on evaluation and management. There is limited research on the effectiveness of strategies used to prevent dog bite injury. A review of two studies evaluating the effectiveness of education as a method of preventing dog bites (in kindergarten and elementary school children) concluded it is still unclear whether it helps reduce rates of dog bite injury [14]. However, this is most likely due to the lack of follow-up to evaluate the incidence of dog bites after education on appropriate behavior around dogs and its consequences was provided. Therefore, more studies in dog bite prevention are needed and future studies should consider evaluating the incidence of dog bites after the intervention. Another important factor in the risk of dog bites is the breed chosen for a house pet. Breeds of dogs are associated with specific temperament, aggressiveness, strength, and activeness. Larger breeds, failure to neuter a dog and selection of male dogs of specific breeds are also associated with more severe wounds and a higher risk of bite injury [3]. Malinois have high prey drive, a trait that can increase the risk of injuries in younger children if not trained properly. This requires tremendous time, patience, and expertise that many households cannot achieve. Therefore, before welcoming a dog to the family, patients should assess breed characteristics, level of responsibility, and safety.

## Conclusions

Dog bite wounds can be fatal, especially to smaller children. We presented a case where a six-year-old male sustained a deep avulsion injury to the right shoulder with right brachial artery contusion after being bitten by a Belgian Malinois. This type of injury requires expedient wound irrigation to prevent secondary infection and evaluation of deeper structures. Antibiotic prophylaxis using Amoxicillin-Clavulanate, frequent irrigation, and healing by tertiary intention were highly effective in preventing infection and providing satisfactory cosmetic outcomes. Further research is needed to find effective educational interventions in reducing the incidence of dog bites and to determine the clinical indications for wound exploration to assess for vascular injury.
